# New roles of N6-methyladenosine methylation system regulating the occurrence of non-alcoholic fatty liver disease with N6-methyladenosine-modified MYC

**DOI:** 10.3389/fphar.2022.973116

**Published:** 2022-08-31

**Authors:** Wenli Cheng, Min Li, Luyun Zhang, Cheng Zhou, Susu Yu, Xinyue Peng, Wenji Zhang, Wenjuan Zhang

**Affiliations:** ^1^ Department of Public Health and Preventive Medicine, School of Medicine, Jinan University, Guangzhou, China; ^2^ Guangdong Provincial Engineering and Technology Research Center for Tobacco Breeding and Comprehensive Utilization, Crops Research Institute, Guangdong Academy of Agricultural Science, Guangzhou, China

**Keywords:** non-alcoholic fatty liver disease (NAFLD), non-alcoholic steatohepatitis (NASH), N6-methyladenosine (m6A), RNA methylation, MYC

## Abstract

Non-alcoholic fatty liver disease (NAFLD) has become a major chronic disease in contemporary society, affected by N6-methyladenosine (m6A) RNA methylation, one of the most common RNA modifications. Compared with healthy control, m6A RNA methyltransferase 3 (METTL3) and METTL14 increased, while Wilms tumor 1-associated protein (WTAP) and RNA-binding motif protein 15 (RBM15) decreased significantly in NAFLD, and the m6A demethylases fat mass and obesity-associated protein (FTO) elevated. Meanwhile, the m6A binding proteins, YT521-B homology (YTH) domain-containing 1 (YTHDC1), YTHDC2, insulin-like growth factor 2 mRNA binding protein 1 (IGF2BP1), heterogeneous nuclear ribonucleoprotein C (HNRNPC), and HNRNPA2B1 were decreased, while eukaryotic translation initiation factor 3 subunit H (EIF3H) was increased significantly. All these changes of m6A regulators had significant differences between healthy control and NAFLD, but no differences between the NAFL and NASH group. The expression level of RBM15, HNRNPC, and HNRNPA2B1 were related to body fat index. RBM15, YTHDC2, HNRNPC, HNRNPA2B1, and EIF3H were related to steatosis. Also, KIAA1429 and YTH domain family 1 (YTHDF1) were related to lobular inflammation. Taken together, m6A regulators were involved in the occurrence of NAFLD. More importantly, abnormal MYC was determined as a key link to m6A regulation of NAFLD. The higher MYC mRNA level was accompanied by higher HDL cholesterol and unsaturated fatty acid proportions, as well as lower fat mass, glucose, and transaminase. Taken together, dysregulation of m6A methylation caused steatosis and fibrosis, affecting the occurrence of NAFLD, and MYC might be its potential target.

## Introduction

Because of the rapid development of the economy and the transformation of lifestyles, non-alcoholic fatty liver disease (NAFLD), as a common chronic metabolic disease, has become a major concern for human healthcare and may result in increased mortality and morbidity on progressing to non-alcoholic steatohepatitis (NASH), cirrhosis, or even hepatocellular carcinoma (Zhang et al., 2022). However, the pathophysiological mechanism of NAFLD has not been fully elucidated up to now and there is still a lack of effective and specific therapeutic drugs in clinical practice (Loomba et al., 2021). Therefore, the pathogenesis of NAFLD and the potential intervention targets have become the hotspots in the research field of liver and metabolism.

N6-methyladenosine (m6A) methylation, as a post-transcriptional RNA epigenetic modification, is catalyzed at the N6 site of adenine by RNA methyltransferases (Desrosiers et al., 1974). m6A methylation is dynamically reversible, regulated by RNA methyltransferases, demethylases, and binding proteins. m6A methyltransferases act as “writers” to promote m6A methylation in RNA, including the methyltransferase 3 (METTL3) (Bokar et al., 1997), METTL14 (Liu et al., 2014), Wilms tumor 1-associated protein (WTAP) (Agarwala et al., 2012), KIAA1429 (Schwartz et al., 2014), RNA-binding motif protein 15/15B (RBM15/15B) (Patil et al., 2016), zinc finger CCCH domain-containing protein 13 (ZC3H13) (Wen et al., 2018), and Cbl proto-oncogene like 1 (CBLL1) (Bawankar et al., 2021). The m6A demethylases act as “erasers” to eliminate m6A methylation in RNA, including fat mass and obesity-associated protein (FTO) (Jia et al., 2011) and alkB homolog 5 (ALKBH5) (Zheng et al., 2013). The m6A binding proteins act as “readers” to recognize the m6A motif and mediate the m6A biological processes, including YT521-B homology (YTH) domain-containing 1/2 (YTHDC1/2), YTH domain family 1/2/3 (YTHDF1/2/3) (Zhu et al., 2014; Liao et al., 2022), insulin-like growth factor 2 mRNA binding protein 1/2/3 (IGF2BP1/2/3) (Huang et al., 2018), heterogeneous nuclear ribonucleoprotein A2/B1 (HNRNPA2B1) (Alarcón et al., 2015), HNRNPC (Liu et al., 2015), and eukaryotic translation initiation factor 3 subunit H (EIF3H) (Choe et al., 2018). RNA methyltransferases, demethylases, and binding proteins work together, mediating m6A methylation to regulate the stability, localization, transportation, splicing, and translation of mRNA, and then eventually affect the fate of mRNA molecules closely related to the biological structure and function of the organism (Jiang et al., 2021).

Recently, more and more evidence showed that m6A methylation was related to the occurrence and development of NAFLD (Li et al., 2021; Qin et al., 2021; Zhou et al., 2021). However, the mechanism of m6A methylation regulating NAFLD and its targeted RNAs are still unclear.

It was well-known that lipid deposition, inflammation, and fibrosis were the troikas of NAFLD. In this study, we analyzed the correlations between the expression of m6A regulators and lipid deposition, inflammation, fibrosis, and immune infiltration in NAFLD, and proposed a new hypothesis for the potential therapeutic targets of m6A regulation in NAFLD.

## Materials and methods

### Gene expression datasets and clinical information

Through careful screening, the GSE89632 dataset of Gene Expression Omnibus (GEO) not only contained expression profiling of multiple genes but also had detailed groups by the non-alcoholic fatty liver (NAFL), NASH, and healthy control, consistently with the design. Meanwhile, it covered a wide range of patients’ clinical characteristics, including lipid deposition, inflammation, and fibrosis. From the GSE89632 dataset, the gene expression data of 63 samples were collected with human liver biopsy tissues, including 20 NAFL, 19 NASH, and 24 healthy controls. The probe ID was converted into gene symbols by the annotation files GPL14951-11332 and the mean value of the same gene was extracted.

The clinical information of samples was also extracted from GSE89632 datasets to analyze the association between the expression of m6A regulators and anthropometric as well as biochemical variables of the samples, including BMI, waist, steatosis percentage, lobular inflammation, and fibrosis.

A single-cell RNA sequencing dataset, GSE115469, was included to make up the human liver and its immune microenvironment.

The datasets GSE164760, GSE37031, GSE48452, and GSE63067 were merged, which consisted of 186 samples for validation to avoid the potential bias, including 125 patients and 61 healthy controls. The package limma (version 3.50.1) was used to adjust for the batch effect.

### Immune infiltration scores and the relevance

The single-cell RNA sequencing dataset GSE115469 provided a map of the human hepatic immune microenvironment by representing in a two-dimensional t-distributed stochastic neighbor embedding (tSNE) plane and clusters were identified *via* the Seurate package (version 4.1.1). Also, the Human Primary Cell Atlas data were used to annotate individual cell types by the SingleR package (version 1.8.1).

Using the single-cell RNA sequencing dataset GSE115469 as a reference, the cell fraction of each sample in GSE89632 was calculated by CIBERSORTx, which is a suite of machine learning tools for the assessment of cellular abundance and cell type-specific gene expression patterns from bulk tissue transcriptome profiles (Steen et al., 2020). The samples with significance were filtered in and formed a matrix of cell relative percentage data. The differences in cell components among healthy and NAFLD were compared. Also, the correlations were analyzed between the m6A regulators and immune cell percent.

### Exploration of the target pathways and genes in N6-methyladenosine methylation

The coexpression relationships were analyzed among m6A regulators. The m6A regulators with high correlation and cluster were thought to be collaborative m6A regulators to coregulate m6A methylation.

The samples were divided into high and low expression groups according to the median of different collaborative m6A regulators to conduct Gene Set Enrichment Analysis (GSEA) (Subramanian et al., 2005) and detect differentially expressed genes (DEGs), respectively. The intersections of results of GSEA and DEGs were visualized by Venn diagrams.

GSEA was performed by the clusterProfiler R package (version 4.2.2) according to the Kyoto Encyclopedia of Genes and Genomes (KEGG). Genes with |log2 fold change (logFC)| > 1 and the result of Wilcox test *p* < 0.05 were considered as DEGs by using the limma package (version 3.50.1). The common signaling pathway and DEGs were considered the potential targets of m6A methylation.

### Protein–protein interaction (PPI) network construction

PPI networks were constructed by STRING online (https://cn.string-db.org/cgi/input.pl) to analyze the interaction among the collaborative m6A regulators and DEGs. Confidence >0.400 was used as the filter condition. The PPI network hid disconnected proteins and was visualized by STRING.

### Statistical analysis

The *t*-test was performed to access the expression difference between NAFLD and healthy control. The ANOVA was performed to the difference among the multiple clinical stages. Turkey’s post hoc test was used for comparison between groups. The correlations within the continuous variables were analyzed by Pearson’s test, while the discrete variables were analyzed by Spearman’s test. All calculations in the study were dependent on R language (version 4.4.1) and Microsoft Excel for Mac (version 16.59). The statistical significance was defined as a two-sided *p* < 0.05.

The *t*-test was performed to access the expression difference between NAFLD and healthy control. The ANOVA was performed to the difference among the multiple clinical stages. Turkey’s post hoc test was used for comparison between groups.

The waist, body mass index (BMI), and steatosis percentage belonged to the continuous variables, while inflammation and fibrosis were the discrete variables statistically. The correlations within the continuous variables were analyzed by Pearson’s test, while the discrete variables were analyzed by Spearman’s test. In addition, the correlation between different gene expression levels and the correlations between m6A regulators and cell percentage were both analyzed by Pearson’s test. Also, the last, the relationships between MYC expression and clinical characteristics were all analyzed by Pearson’s test except for fibrosis, lobular inflammation, and ballooning, which were discrete variables. All calculations in the study were dependent on R language (version 4.4.1) and Microsoft Excel for Mac (version 16.59). The statistical significance was defined as a two-sided *p* < 0.05.

## Results

### Overview of expression for N6-methyladenosine regulators in non-alcoholic fatty liver disease

According to the current research and cognition, there are 25 m6A regulators as known, including nine writers, such as METTL3, METTL14, WTAP, KIAA1429, RBM15/15B, ZC3H13, CBLL1, and METTL16, two erasers of FTO and ALKBH5, and 14 readers, such as YTHDC1/2, YTHDF1/2/3, IGF2BP1/2/3, HNRNPC, HNRNPG, HNRNPA2B1, EIF3H, PRCC2A, and FMRP (Wang et al., 2022). A total of 21 m6A regulators were common and could be detected in the genome-wide expression dataset, including METTL3, METTL14, WTAP, KIAA1429, RBM15/15B, ZC3H13, CBLL1, FTO, ALKBH5, YTHDC1/2, YTHDF1/2/3, IGF2BP1/2/3, HNRNPC, HNRNPA2B1, and EIF3H separately, which hence were extracted and formed an expression matrix to compare their levels between the healthy controls and NAFLD samples.

**FIGURE 1:**
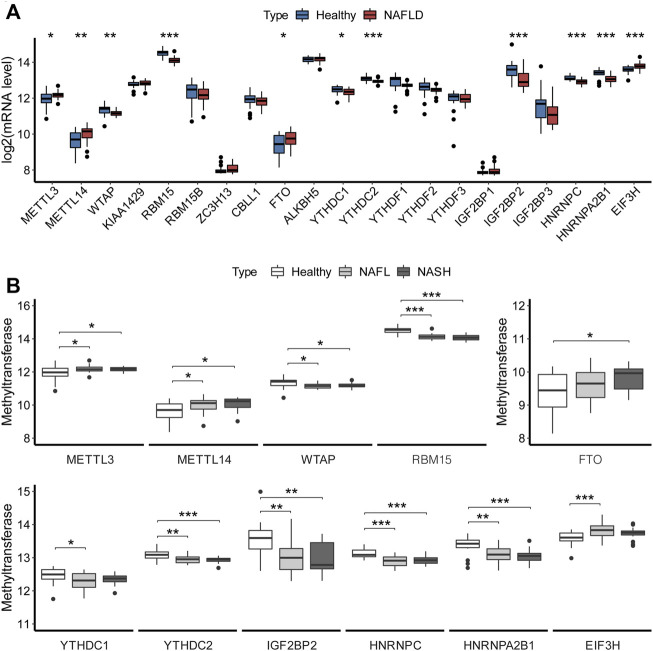
Overview of expression of m6A regulators in NAFLD. **(A)** Expression levels of m6A regulators in liver tissues. The *t*-test analyzed the difference in the expression levels of m6A regulators between the healthy and NAFLD tissues. **(B)** Boxplot showed the association of expression with clinical stages for m6A regulators. Turkey’s post hoc test was used for comparison between groups to analyze the difference in the expression of m6A regulators among different clinical stages of NAFLD. The results of significant difference analysis: *p < 0.05; **p < 0.01; and ***p < 0.001.

We compared the expression of m6A regulators between healthy controls and NAFLD samples. As shown in Figure 1A, compared with the controls, the expressions of m6A regulators increased significantly (*p* < 0.05), including METTL3 (logFC = 0.237), METTL14 (logFC = 0.377), FTO (logFC = 0.300), and EIF3H (logFC = 0.193), while some other m6A regulators significantly decreased, including WTAP (logFC = − 0.385), RBM15 (logFC = − 0.396), YTHDC1 (logFC = − 0.172), YTHDC2 (logFC = − 0.148), IGF2BP2 (logFC = − 0.514), HNRNPC (logFC = − 0.209), and HNRNPA2B1 (logFC = − 0.414) in NAFLD. Others had no significant changes, including KIAA1429, RBM15B, ZC3H13, CBLL1, ALKBH5, YTHDF1, YTHDF2, YTHDF3, IGF2BP1, and IGF2BP3 between NAFLD and control group. In general, compared with control, the expression levels of METTL3 and METTL14 increased, while WTAP and RBM15 decreased in NAFLD. The m6A demethylases FTO elevated. To the m6A binding proteins, YTHDC1, YTHDC2, IGF2BP1, HNRNPC, and HNRNPA2B1 were decreased, while EIF3H was increased in NAFLD. The most expression levels of m6A binding proteins were decreased in NAFLD. It might suggest that m6A modification was generally downregulated in NAFLD.

Also, the expression levels of 11 m6A regulators with significant differences between the healthy control and NAFLD samples were further assessed for the different clinical stages of NAFLD, as shown in Figure 1B.

Then, the expression levels of 11 significantly differential m6A regulators were assessed for the different clinical stages of NAFLD, as shown in Figure 1B. The METTL3, METTL14, WTAP, and RBM15 changed significantly between the control and NAFL, as well as control and NASH, without the significant changes between NAFL and NASH. But the levels of METTL3 and METTL14 were increased in NAFLD, while the WTAP and RBM15 were decreased. FTO had a significant increase between control and NASH. And RNA methylation binding proteins also have significantly changed between control and NAFL, as well as control and NASH, but without significant changes between NAFL and NASH, which were almost downregulated in NAFLD, except for EIF3H. It might imply that m6A modification acted only at the occurrence stage of NAFLD, not in the course of the progression.

### Correlation of levels for N6-methyladenosine regulators with body fat indexes

**FIGURE 2:**
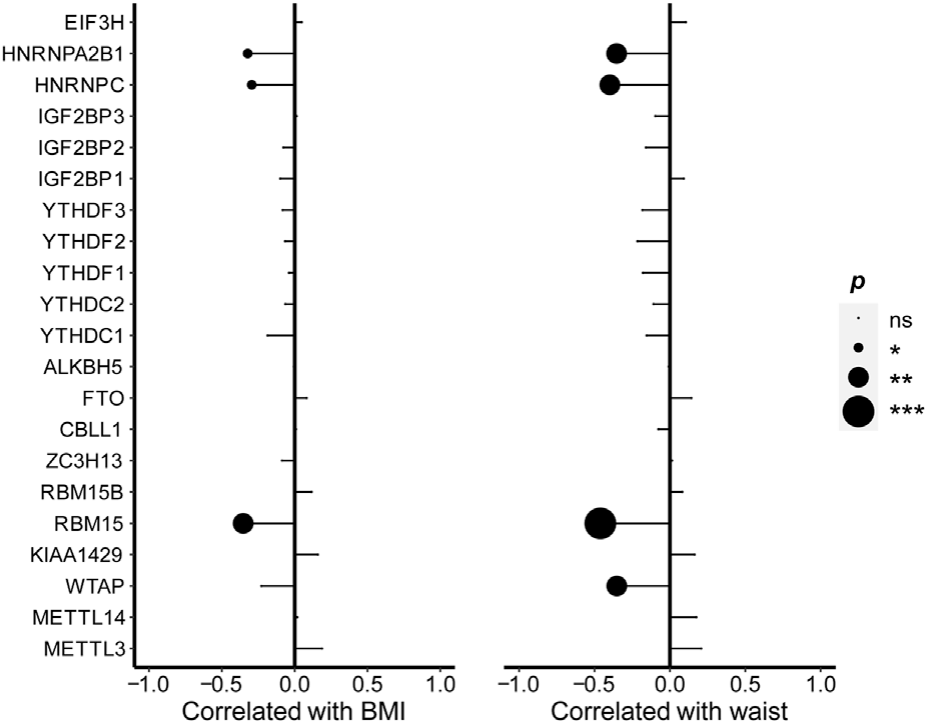
Correlations between the expression of m6A regulators and BMI as well as waist. Pearson’s correlation was performed to calculate the correlation between the expression of m6A regulators and BMI as well as waist. The results of significant difference analysis: ns ≥ 0.05; *p < 0.05; **p < 0.01; and ***p < 0.001.

From Figure 2, the correlation analysis was shown to examine the associations between the expression of m6A regulators and body fat indexes, respectively, including BMI and waist. The WTAP expression was negatively correlated with the waist. The expression of RBM15 (*r* = − 0.353, *p* = 0.005), HNRNPC (*r* = − 0.295, *p* = 0.021), and HNRNPA2B1 (*r* = − 0.323, *p* = 0.011) was negatively related to BMI separately. The expression of WTAP (*r* = − 0.352, *p* = 0.007), RBM15 (*r* = − 0.461, *p* < 0.001), HNRNPC (*r* = − 0.398, *p* = 0.002), and HNRNPA2B1 (*r* = − 0.353, *p* = 0.007) was negatively related to waist.

The expression levels for RBM15, HNRNPC, and HNRNPA2B1 were negatively correlated with BMI and waist and seemed to be sensitive indicators of body fat in NAFLD patients.

### Correlation of levels for N6-methyladenosine regulators with hepatic steatosis, inflammation, and fibrosis

**FIGURE 3:**
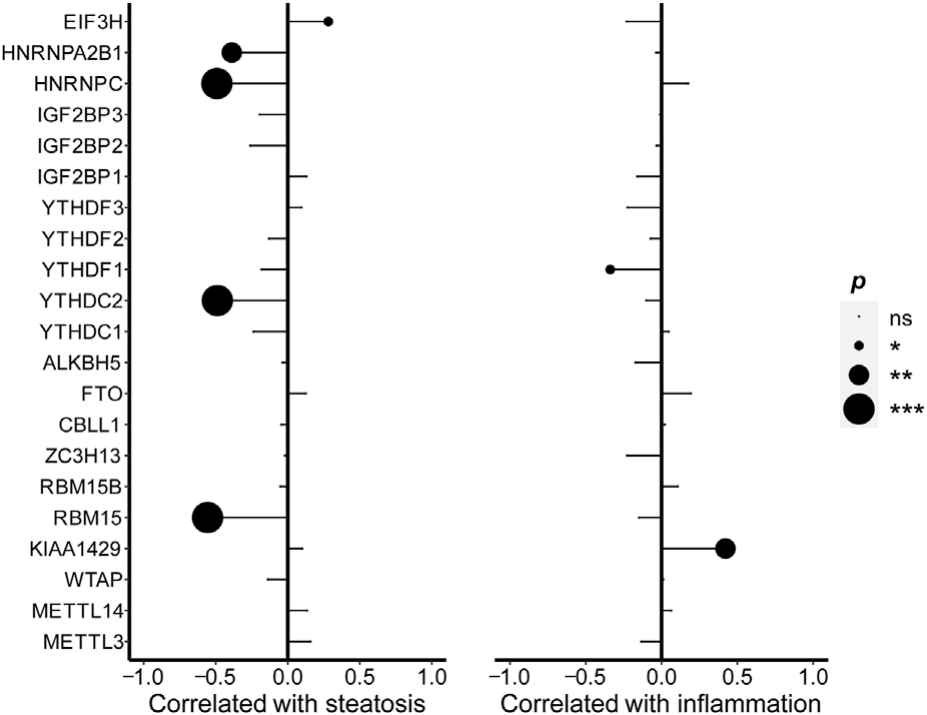
Correlations between m6A regulators and steatosis as well as inflammation. Pearson’s correlation was performed to calculate the correlation between the expression of m6A regulators and hepatic steatosis. Spearman’s correlation was performed to calculate the correlation between the expression of m6A regulators as well as lobular inflammation. The results of significant difference analysis: ns ≥ 0.05; *p < 0.05; **p < 0.01; and ***p < 0.001.

The correlation analysis was also performed to examine the association between the expression of m6A regulators and steatosis percentage. The expression level of EIF3H was positively related to steatosis percentage (*r* = 0.282, *p* = 0.035), while the expressions of RBM15 (*r* = − 0.557, *p* < 0.001), YTHDC1 (*r* = − 0.238, *p* = 0.078), YTHDC2 (*r* = − 0.489, *p* < 0.001), HNRNPC (*r* = − 0.492, *p* < 0.001), and HNRNPA2B1 (*r* = − 0.389, *p* = 0.003) were negatively related to steatosis percentage as shown in Figure 3. The expression level of KIAA1429 was positively related to the severity degree of lobular inflammation (*r* = 0.422, *p* = 0.008), while YTHDF1 was negatively related to the severity degree of lobular inflammation (*r* = − 0.339, *p* = 0.038). But there was no significant relationship between the expression level of m6A regulators and liver fibrosis.

### Correlations of levels of N6-methyladenosine regulators with immune infiltration by combination with a single-cell dataset

**FIGURE 4:**
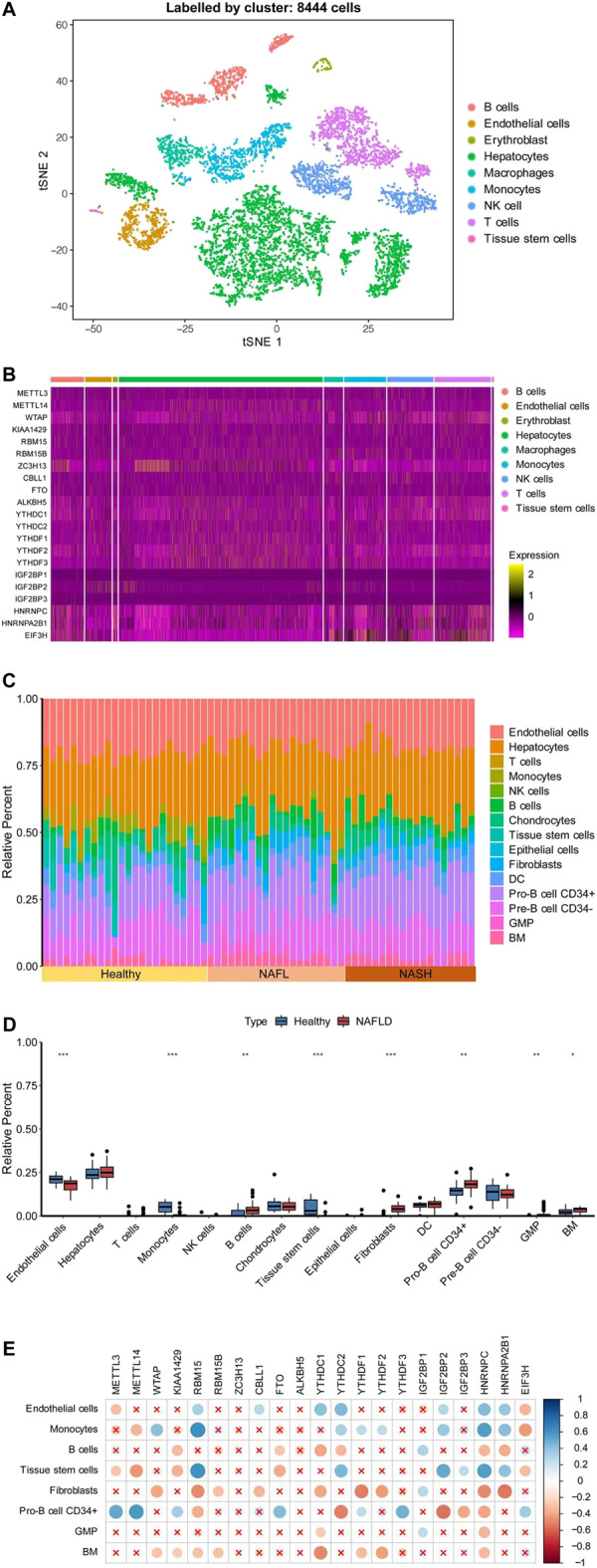
Correlations of levels of m6A regulators with immune infiltration by combination with a single-cell dataset. **(A)** Distinct cell clusters were revealed in healthy human liver. **(B)** Heatmap showed the expression of the m6A regulator in each cell. **(C)** Histogram showed the relative percentage of different types of cells in each sample of GSE89632. **(D)** t-tests were performed to analyze the difference in cell percent between healthy control and NAFLD. The results of significant difference were showed as *p < 0.05; **p < 0.01; or ***p < 0.001. **(E)** Pearson’s test was conducted to analyze the correlations of the m6A regulators’ expression level with the cell percent. The ratio of correlation was shown in color. Blue was a positive correlation and red was negative correlation. Color darker, circle bigger implied a stronger correlation. Demerit marks showed *p* > 0.05.

To evaluate whether m6A regulators were involved in the immune responses of NAFLD more accurately, we collected a large sample from GSE115469, a dataset of single-cell RNA sequencing of the human liver. A total of 8,444 cells were collected from the liver, which was distinct and identified for each cluster in PCA (principal component analysis) as shown in Figure 4A. The cell clusters in the liver mainly included B-cells, endothelial cells, erythroblasts, hepatocytes, macrophages, monocytes, NK cells, T-cells, and tissue stem cells. The largest cluster of cells was the hepatocytes. As shown in Figure 4B, except for IGFBP1/2/3, the expressions of m6A regulators were all detected in each cluster. Furthermore, based on the CIBERSORTx algorithm, the genome-wide expression data GSE89632 and the single-cell sequencing data GSE115469 were combined. The cell annotations of single-cell sequencing data as reference were taken to calculate the immune cell composition of each sample in genome-wide expression as shown in Figure 4C. The components of endothelial cells, hepatocytes, T-cells, monocyte, NK cells, B-cells, chondrocytes, tissue stem cells, epithelial cells, fibroblasts, dendritic cells (DC), pro-B cell CD34^+^, pre-B cell CD34^−^, granulocyte monocyte progenitor (GMP), and bone marrow (BM) were recognized and calculated in each sample as shown in Figure 4D. Interestingly, the compositions of endothelial cells and hepatocytes were the majority in each sample and the proportions of cell composition were different under the specific pathological conditions. The B-cell, fibroblasts, Pro-B cell CD34^+^, GMP, and BM components were significantly higher in NAFLD than those in healthy liver separately, while the endothelial cells, monocytes, and tissue stem cells were lower relatively. Therefore, m6A regulators were related to the component of immune cells and suggested being involved in the immune infiltration of NAFLD.

### The coexpression relationship of N6-methyladenosine regulators as well as the target genes and the related enrichment pathways

**FIGURE 5:**
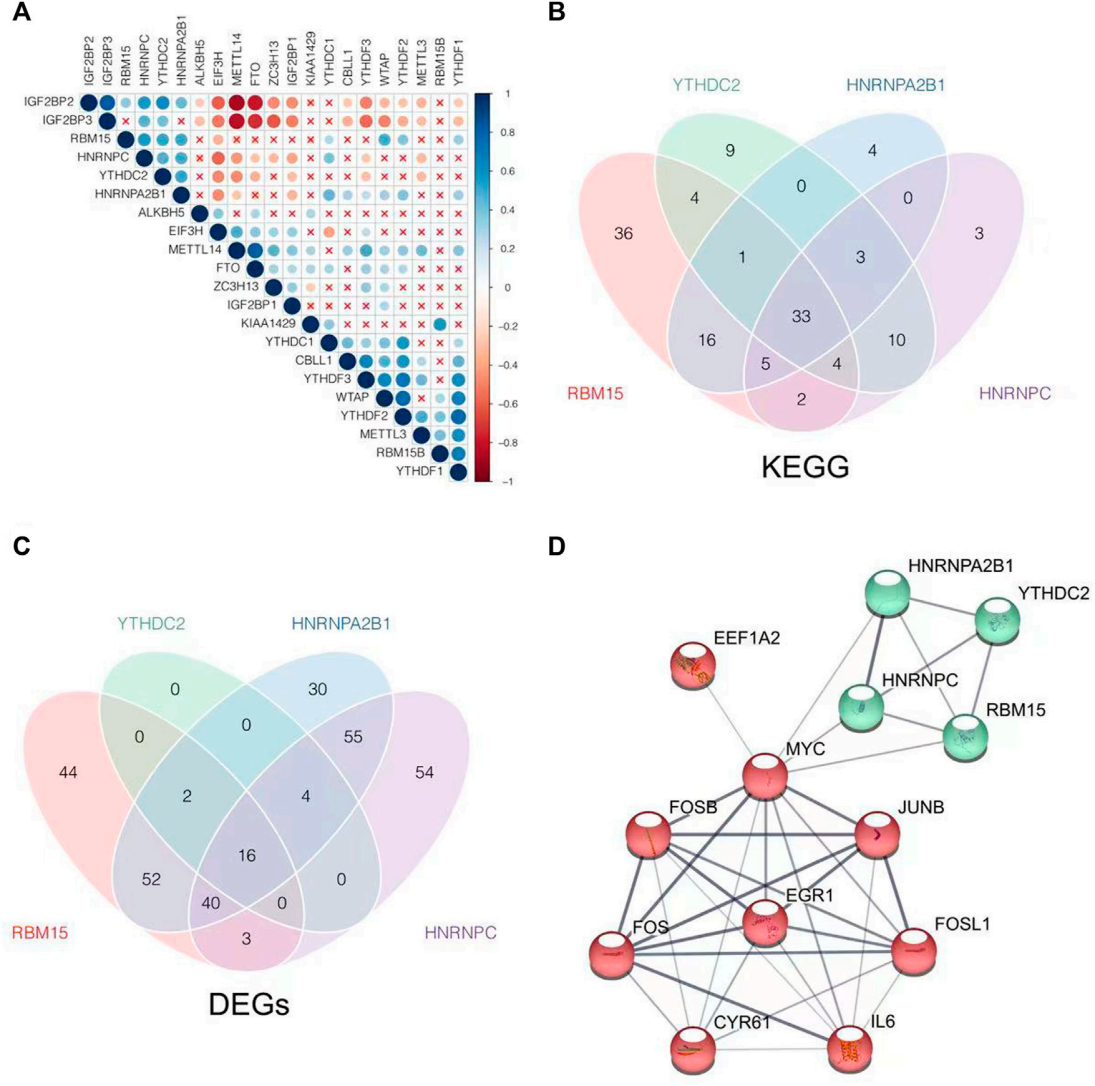
Explore the coexpression relationship of m6A regulators as well as the target genes and enrichment pathways of m6A related. **(A)** Pearson’s test was conducted to analyze the coexpression relationship of m6A regulators. **(B)** Intersection of KEGG enrichment pathways, which were analyzed by GSEA according to stratify by the median expression level of RBM15, YTHDC2, HNRNPA2B1, and HNRNPC, was visualized as a Venn diagram. **(C)** Intersection of DEGs which were screened according to stratify by the median expression level of RBM15, YTHDC2, HNRNPA2B1, and HNRNPC, was visualized as a Venn diagram. **(D)** PPI network was construed by common DEGs as well as RBM15, YTHDC2, HNRNPA2B1, and HNRNPC, with the disconnected proteins being hidden. Wider lines indicated stronger evidence of protein interaction.

The coexpression relationships of m6A regulators were evaluated further. There was a strong coexpression relationship among RBM15, HNRNPC, YTHDC2, and HNRNPA2B1 as shown in Figure 5A. The m6A regulators with high correlation and cluster were considered to be collaborative components to coregulate m6A RNA methylation jointly. Therefore, their intersection of GSEA and DEGs was more likely to be the actual effects of the m6A methylation rather than the individual m6A regulator. They did act on the m6A methylation collectively. GESA was performed to identify the differences in biological processes between high and low expression groups of RBM15, HNRNPC, YTHDC1, and HNRNPA2B1. The 33 KEGG pathways were significantly enriched in each group as shown in Figure 5B. The 33 KEGG pathways included steroid hormone biosynthesis, cytochrome P450, nucleotide metabolism, and signaling pathways of mitogen-activated kinase-like protein (MAPK), phosphatidylinositol 3-kinase (PI3K)/Akt, Janus kinase (JAK)/signal transducer and activator of transcription (STAT), interleukin 17 (IL-17), tumor necrosis factor (TNF), and so on, as shown in Table 1.

**TABLE 1:** Gene set enrichment analysis of m6A regulators.

KEGG pathway	RBM15		YTHDC2		HNRNPC		HNRNPA2B1	
	NES	p	NES	p	NES	p	NES	p
Steroid hormone biosynthesis	−2.046	<0.001	−1.709	0.010	−1.938	0.002	−1.669	0.030
Oxidative phosphorylation	−1.969	<0.001	−1.958	<0.001	−1.618	0.011	−1.485	0.038
Metabolism of xenobiotics by cytochrome P450	−2.029	<0.001	−1.602	0.029	−1.825	0.003	−1.693	0.019
Drug metabolism—cytochrome P450	−2.312	<0.001	−1.705	0.011	−2.023	<0.001	−1.893	0.002
Drug metabolism—other enzymes	−1.925	<0.001	−1.668	0.012	−1.751	0.006	−1.800	0.005
Nucleotide metabolism	−1.998	<0.001	−1.933	<0.001	−1.720	0.008	−1.865	0.002
Biosynthesis of cofactors	−1.970	<0.001	−1.940	<0.001	−1.888	<0.001	−1.495	0.036
Platinum drug resistance	−1.543	0.016	−1.591	0.029	−1.569	0.032	−1.558	0.038
Ribosome	−1.924	<0.001	−2.551	<0.001	−2.259	<0.001	−1.997	<0.001
DNA replication	−1.623	0.033	−1.853	0.007	−1.850	0.008	−2.076	0.001
Base excision repair	−1.746	0.011	−2.099	<0.001	−1.980	0.003	−2.044	0.001
Nucleotide excision repair	−1.836	0.005	−2.221	<0.001	−2.033	<0.001	−1.788	0.013
Fanconi anemia pathway	−1.573	0.037	−1.612	0.029	−1.875	0.003	−1.664	0.036
MAPK signaling pathway	1.749	<0.001	1.592	0.002	1.503	0.010	1.600	0.001
Cytokine–cytokine receptor interaction	2.188	<0.001	1.835	<0.001	2.090	<0.001	1.983	<0.001
Viral protein interaction with cytokine and cytokine receptor	2.212	<0.001	1.483	0.050	1.889	0.001	1.989	<0.001
Lysosome	−1.502	0.013	−1.570	0.022	−1.539	0.021	−1.502	0.032
Peroxisome	−2.263	<0.001	−2.218	<0.001	−2.067	<0.001	−1.933	0.001
PI3K-Akt signaling pathway	1.639	<0.001	1.509	0.002	1.534	0.002	1.504	0.002
Osteoclast differentiation	2.306	<0.001	1.795	0.002	1.728	0.003	1.984	<0.001
JAK-STAT signaling pathway	2.268	<0.001	1.875	<0.001	2.047	<0.001	2.043	<0.001
IL-17 signaling pathway	2.589	<0.001	2.156	<0.001	2.253	<0.001	2.473	<0.001
TNF signaling pathway	2.604	<0.001	1.894	<0.001	1.844	0.001	2.261	<0.001
Thermogenesis	−1.726	<0.001	−1.887	<0.001	−1.808	<0.001	−1.397	0.033
Olfactory transduction	2.113	<0.001	3.014	<0.001	3.072	<0.001	2.743	<0.001
AGE-RAGE signaling pathway in diabetic complications	2.232	<0.001	1.934	<0.001	1.749	0.005	2.028	<0.001
Alcoholism	−1.578	0.004	−1.988	<0.001	−2.045	<0.001	−2.179	<0.001
Amoebiasis	1.903	<0.001	1.722	0.004	1.762	0.003	1.740	0.006
MicroRNAs in cancer	1.706	<0.001	1.762	<0.001	1.845	<0.001	1.732	<0.001
Bladder cancer	1.973	0.001	1.725	0.025	1.761	0.015	1.739	0.026
Systemic lupus erythematosus	−1.604	0.005	−2.393	<0.001	−2.476	<0.001	−2.562	<0.001
Rheumatoid arthritis	2.294	<0.001	1.654	0.014	1.839	0.002	1.876	0.001
Hypertrophic cardiomyopathy	1.543	0.024	1.695	0.008	1.746	0.007	1.558	0.038

*NES, normalized enrichment score; P, adjusted P. The adjustment method was Benjamini & Hochberg.

Then, the 16 common DEGs were obtained by the expression level stratification in RBM15, HNRNPC, YTHDC2, and HNRNPA2B1 as shown in Figure 5C. Through the PPI network construction, we found that MYC was closely connected with the common DEGs to the m6A regulators of RBM15, HNRNPC, YTHDC2, and HNRNPA2B1 as shown in Figure 5D. MYC might be the key link to be involved in m6A methylation during NAFLD disease progression.

### MYC expression profile in non-alcoholic fatty liver disease

**FIGURE 6:**
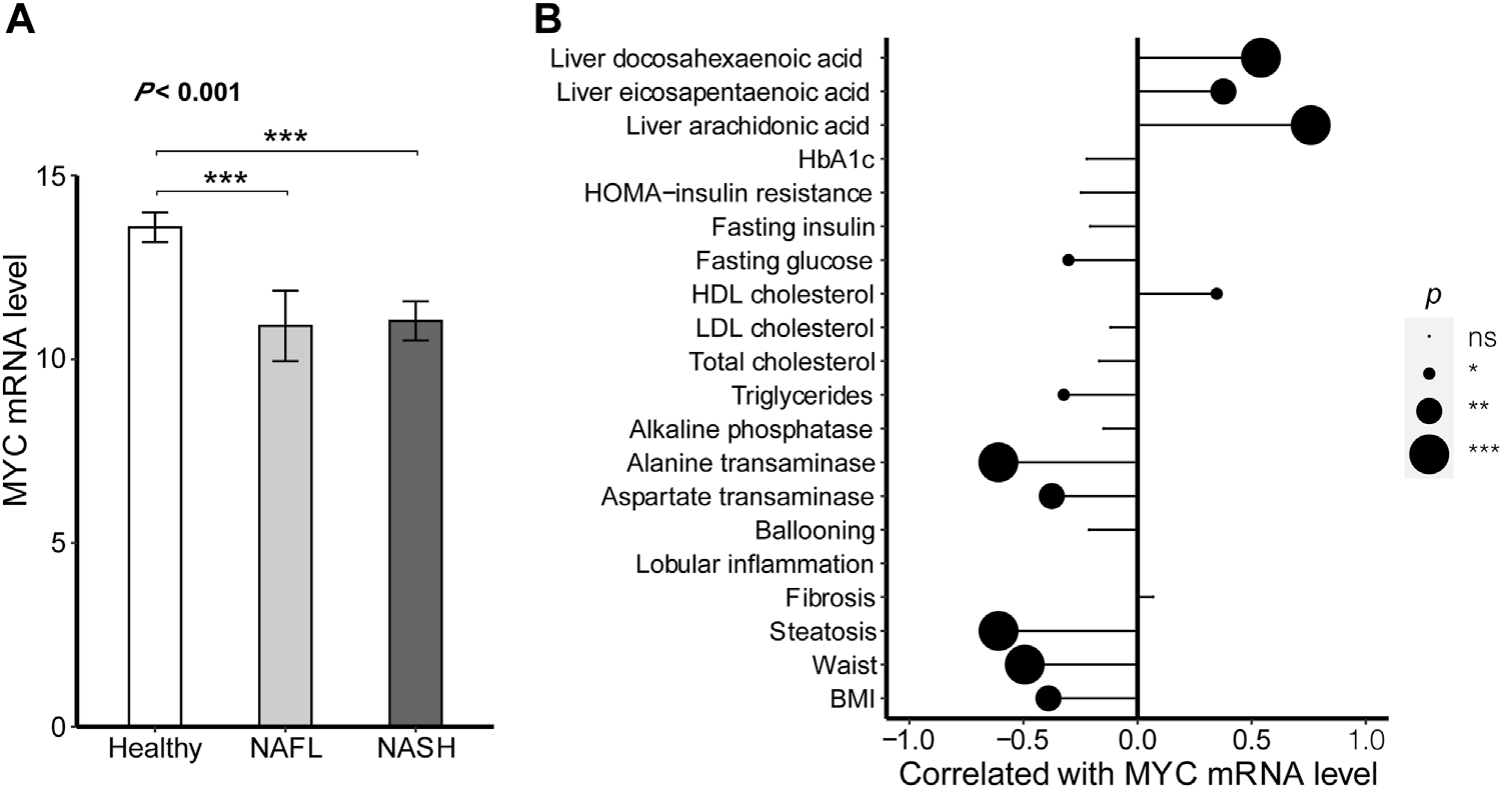
MYC expression profile in NAFLD. **(A)** Difference was analyzed in the expression of MYC between different clinical stages of NAFLD. The ANOVA was performed to the difference among the multiple clinical stages. Turkey’s post hoc test was used for comparison between groups. **(B)** Correlations between the expression of MYC and patients’ clinical characteristics. The correlations were all analyzed by Pearson’s test except for fibrosis, lobular inflammation, and ballooning. The correlations between the MYC expression and the characteristic of fibrosis, lobular inflammation, and ballooning were conducted by Spearman’s test. The results of significant difference analysis: ns ≥ 0.05; *p < 0.05; **p < 0.01; and ***p < 0.001.

In order to verify whether MYC was related to NAFLD diseases, its expression level was assessed in healthy, NAFL, and NAFLD livers. As shown in Figure 6A, the MYC expression levels were differential among the different clinical stages of liver diseases. Compared with the healthy samples, the mRNA levels for MYC were lower in both NAFL (*p* < 0.001) and NASH (*p* < 0.001), while the difference in MYC expression between NAFL and NASH had no significance (*p* = 0.802).

Meanwhile, the associations between MYC mRNA levels and patients’ clinical characteristics were also assessed as shown in Figure 6B. The MYC expression was positively related to HDL cholesterol (*r* = 0.347, *p* = 0.012) and the proportion of unsaturated fatty acids in liver lipids including arachidonic acid (*r* = 0.759, *p* < 0.001), eicosapentaenoic acid (*r* = 0.376, *p* = 0.006), and docosahexaenoic acid (*r* = 0.540, *p* < 0.001). However, the MYC expression was negatively related to BMI (*r* = − 0.609, *p* < 0.001), waist (*r* = − 0.494, *p* < 0.001), steatosis (*r* = − 0.609, *p* < 0.001), triglycerides (*r* = − 0.324, *p* = 0.017), fasting glucose (*r* = − 0.303, *p* = 0.022), and transaminase including aspartate transaminase (*r* = − 0.376, *p* = 0.003) and alanine transaminase (*r* = − 0.610, *p* < 0.001). The higher MYC mRNA level was accompanied with the higher HDL cholesterol and unsaturated fatty acid proportions as well as the lower fat mass, glucose, and transaminase, but without significance in indicators of inflammation and fibrosis. These findings suggest that the MYC might be associated with NAFLD by obesity rather than inflammatory response.

### Verification by another dataset

**FIGURE 7:**
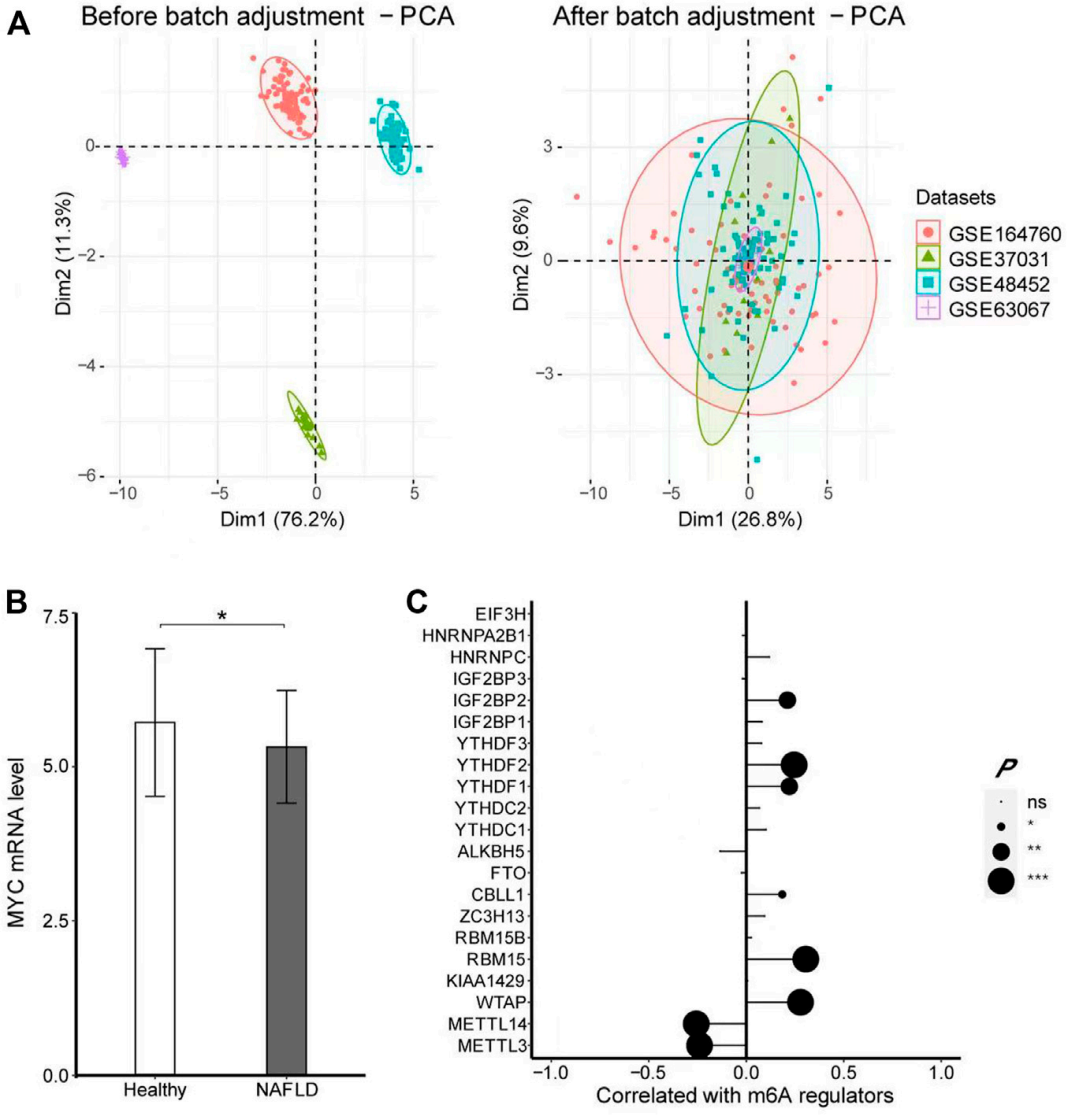
MYC expression profile in NAFLD by using four datasets. **(A)** PCA showed the datasets of GSE164770, GSE37031, GSE48452, and GSE63067 were merged and adjusted in batches. **(B)** -test was conducted to analyze the difference in the expression of MYC between healthy controls and NAFLD. **(C)** Pearson’s test was performed to analyze the correlation between MYC and m6A regulators. The results of significant difference analysis: ns ≥ 0.05; *p < 0.05; **p < 0.01; and ***p < 0.001.

To verify whether the expression of MYC was indeed decreased in NAFLD patients, we adopted another four datasets, including GSE164760, GSE37031, GSE48452, and GSE63067 with 186 samples in all to validate the results further, which contained 125 NAFLD patients and 61 healthy controls, merged and batch-adjusted as shown in Figure 7A. There was a significant decrease in MYC level in NASH (logFC = − 0.394, *p* = 0.026) as shown in Figure 7B. In addition, the expression level of MYC was negatively correlated with METTL3 (*r* = − 0.239, *p* < 0.001) and METTL14 (*r* = − 0.257, *p* < 0.001) with significance, while positively correlated with WTAP (*r* = 0.278, *p* < 0.001), RBM15 (*r* = 0.305, *p* < 0.001), CBLL1 (*r* = 0.184, *p* = 0.012), YTHDF1 (*r* = 0.221, *p* = 0.002), YTHDF2 (*r* = 0.245, *p* < 0.001), and IGF2BP2 (*r* = 0.211, *p* = 0.004) in the merged datasets as shown in Figure 7C. These abovementioned results confirmed the hypothesis that MYC expression was decreased in liver tissues of NAFLD patients, associated with m6A regulators closely.

## Discussion

Based on the results, the expression of METTL3, METTL14, WTAP, RBM15, FTO, YTHDC1, YTHDC2, IGF2BP2, HNRNPC, HNRNA2B1, and EIF3H were significantly different between healthy liver and NAFLD with a differential expression profile. Compared with healthy control, the expression levels of METTL3 and METTL14 increased, while WTAP and RBM15 decreased. Also, FTO elevation was consistent with the earlier study (Guo et al., 2013). YTHDC1, YTHDC2, IGF2BP1, HNRNPC, and HNRNPA2B1 were decreased, while EIF3H was increased. The most expression levels of m6A binding proteins were decreased in NAFLD. It might suggest that m6A modification was generally downregulated in NAFLD. The differences in m6A regulators’ expression produced the relative effects in combination with each other, contributing to the phenotypes of NAFLD disease finally. Small differences with statistical significance might form a bio-effect web to play important roles together. In addition, the expressions of m6A regulators have a significant difference between healthy control and NAFLD, with no difference between the NAFL and NASH groups. It might imply that m6A modification acted at the occurrence of NAFLD without the progression.

As known to all, lipid deposition, inflammation, and fibrosis were the troikas of NAFLD. Therefore, we explored the relationships between m6A regulators’ expressions and obesity, steatosis, inflammation, and fibrosis in NAFLD by using the indicators of clinical characteristics of samples in the GSE89632 datasets. In terms of body fat indexes, the expression level of RBM15, HNRNPC, and HNRNPA2B1 were negatively correlated with BMI and waist. To hepatic steatosis, the expression level of EIF3H was positively related to steatosis percentage, while the expressions of RBM15, YTHDC2, HNRNPC, and HNRNPA2B1 were negatively related to steatosis percentage. A study pointed out that YTHDC2 was markedly downregulated in the livers of obese mice and NAFLD patients and its overexpression in the livers of obese mice improved liver steatosis (Zhou et al., 2021), which was consistent with our results that YTHDC2 expression decreased in NAFLD and was related to steatosis. In addition, the expression level of KIAA1429 was positively related to the severity degree of lobular inflammation, while YTHDF1 was negatively related. But there was no significant relationship between the expression level of m6A regulators and liver fibrosis.

Furthermore, immune infiltration was also an important factor in the development of NAFLD disease, affecting the inflammatory response of NAFLD as a key role in the pathogenesis of NAFLD. The CIBERSORTx algorithm was used to calculate cell percentages in different samples and to compare differences in immune cell percentages between healthy controls and NAFLD. The results showed that B-cell, fibroblasts, Pro-B cell CD34^+^, GMP, and BM components were significantly higher in NAFLD than healthy liver, while the endothelial cells, monocytes, and tissue stem cells were lower, suggesting that the occurrence of NAFLD led to the component increase of fibroblasts and BM, while the decrease of endothelial cells and tissue stem cells. Also, B-cell, Pro-B cell CD34^+^, GMP, and monocytes might be involved in the occurrence of NAFLD. Therefore, we conducted the correlation between the expression of m6A regulators and the percentage of different kinds of cells. RBM15, YTHDC1, YTHDC2, HNRNPC, and HNRNPA2B1 were almost significantly positively related to endothelial cells, monocytes, and tissue stem cells, while negative related to B-cell, fibroblasts, pro-B cell CD34^+^, and BM. Thus, m6A regulators were associated with lipid deposition, inflammation, and immune microenvironment of NAFLD, involved in the occurrence of NAFLD.

Then, we found that some m6A regulators have a coexpression relationship, indicating that m6A regulators had a synergistic effect on NAFLD or that m6A modification occurred in NAFLD indirectly. The correlations were strong among RBM15, HNRNPC, YTHDC2, and HNRNPA2B1. The samples were divided into groups according to their median expression levels to find out the common KEGG enrichment pathways, including steroid hormone biosynthesis, cytochrome P450 metabolism, and nucleotide metabolism, and signaling pathways of MAPK, PI3K-Akt, JAK-STAT, IL-17, and TNF, which might be the key molecular mechanisms. It was consistent with the report that steroid synthesis was involved in the pathological process of non-alcoholic fatty liver disease by regulating hepatic lipid production and glucose metabolism (Grossmann et al., 2019).

More importantly, we found that MYC might be the most critical link between m6A methylation and the regulation of NAFLD. The 16 genes including MYC, JunB proto-oncogene (JUNB), IL6, FosB proto-oncogene (FOSB), FOS, FOS like 1 (FOSL1), and early growth response 1 (EGR1) were filtrated as the common DEGs in the groups of RBM15, HNRNPC, YTHDC2, and HNRNPA2B1. A PPI network was constructed by 16 common DEGs and the four m6A regulators, and we found that MYC might be the m6A modification target, regulated by RBM15, HNRNPC, YTHDC2, and HNRNPA2B1, affecting the occurrence of NAFLD.

Thus, we focused on MYC which was a highly pleiotropic transcription factor with broad effects on cell proliferation, metabolism, angiogenesis, apoptosis, adhesion, and differentiation, whose overexpression was usually related to cancer (Dang, 2012). In addition to cancer, MYC was also reported to be involved in the regulation of metabolic diseases, including alcoholic liver disease and NAFLD (Nevzorova et al., 2016), (Shin et al., 2013). Reduced MYC expression increased metabolic activity and reduced cholesterol synthesis in the liver (Hofmann et al., 2015; Luo et al., 2021), while overexpression of MYC led to the development of mild steatohepatitis and fibrosis (Guo et al., 2021).

In addition, MYC has been proven to be an important m6A modification target. The m6A regulators regulated the m6A methylation in MYC, affecting the stability and translation efficiency of MYC, and then regulated the expression of MYC, which was an important mechanism for the occurrence and development of cancers (Wu et al., 2021; Zheng et al., 2022). The m6A regulators of METTL3/5, FTO, IGF2BP1/2/3, YTHDF2 (Einstein et al., 2021; Iaiza et al., 2021; Yang et al., 2021; Chen et al., 2022; Du et al., 2022; Hu et al., 2022; Huang et al., 2022; Liu et al., 2022; Luo and Lin, 2022), and so on had been proven to modulate the mechanism. In our study, compared with the healthy control, MYC mRNA levels were lower in both NAFL and NASH, while no differences were found between NAFL and NASH. This was consistent with trends of the m6A regulators downregulated in NAFLD. The m6A regulators also had no significant change between NAFL and NASH.

Similar to RBM15, HNRNPC, and HNRNPA2B1, MYC expression level significance was also negatively related to obesity. The higher MYC mRNA level along with the higher HDL cholesterol and unsaturated fatty acid proportions. The HDL cholesterol and unsaturated fatty acids could promote lipid metabolism, leading the lower fat mass, glucose, and transaminase.

Therefore, we speculate that m6A has an effect on the occurrence of NAFLD and this effect is likely to be produced by modified MYC. In order to eliminate the bias caused by a single dataset, we included another four datasets for verification. The results also confirmed that MYC was indeed downregulated in NAFLD, significantly associated with multiple m6A regulators.

Our results showed that MYC was associated with obesity and fatty metabolism. However, unlike other findings, MYC expression was decreased in obese and steatosis patients. Similarly, the m6A regulators were more sensitive to steatosis rather than inflammation and fibrosis in NAFLD. The expression levels of m6A methylase RBM15, binding protein YTH family, and MYC decreased in the NAFL stage, and there was a coexpression relationship. Therefore, the dysregulation of m6A methylation caused steatosis and fibrosis affected the occurrence of NAFLD, and MYC might be its potential target.

At present, more and more drugs targeting m6A are being developed with good prospects for clinical application and transformation. STM2457 and FB23/FB23-2 were small-molecular inhibitors of m6A regulators, preventing the development of human acute myeloid leukemia cells *in vitro* by specifically suppressing the activities of METTL3 and FTO, respectively, in recent research (Huang et al., 2019; Yankova et al., 2021). In addition, through screening on a 3D proteome-wide scale and activity evaluation, MV1035 could reduce U87 glioblastoma cell line migration as a new ALKBH5 inhibitor (Malacrida et al., 2020). Furthermore, many traditional Chinese medicines from natural products had been also shown to regulate the m6A modification with therapeutic and healthy benefits. Saikosaponin-d also exhibited antileukemic activity by targeting FTO/m6A signaling in human acute myeloid leukemia cells *in vitro* (Sun et al., 2021). Curcumin and epigallocatechin gallate prevented obesity by reducing ALKBH5 or FTO separately in an m6A-dependent manner (Wu et al., 2018; Chen et al., 2021). Those drugs have possessed therapeutic or preventive potentialities *via* regulating m6A modification and the targeted regulators. Because m6A modification was involved in the occurrence of NAFLD rather than the progression, m6A regulators like RBM15 and YTH family might serve as the potential targets for the prevention and treatment of NAFLD in obese people.

As m6A modification is a common, dynamic, and reversible process, its targeted therapy would be possible to prevent or intervene in the occurrence and development of diseases with a bright foreground, including NAFLD or some cancers. The actual clinical application and potential transformation of m6A modification in NAFLD need to be deeply studied. The new findings of m6A regulation in NAFLD will provide good clues for the prevention and treatment strategy in the future.

## Data Availability

The datasets presented in this study can be found in online repositories. The names of the repository/repositories and accession number(s) can be found in the article/Supplementary Material.

## Glossary

NAFLD: non-alcoholic fatty liver disease
m6A: N6-methyladenosine
METTL3: methyltransferase 3
WTAP: Wilms tumor 1-associated protein
RBM15: RNA-binding motif protein 15
FTO: fat mass and obesity-associated protein
YTHDC1: YT521-B homology domain-containing 1
IGF2BP1: insulin-like growth factor 2 mRNA binding protein 1
HNRNPC: heterogeneous nuclear ribonucleoprotein C
EIF3H: eukaryotic translation initiation factor 3 subunit H
YTHDF1: YTH domain family 1
NASH: non-alcoholic steatohepatitis
GEO: Gene Expression Omnibus
NAFL: non-alcoholic fatty liver
tSNE: t-distributed stochastic neighbor embedding
GSEA: Gene Set Enrichment Analysis
DEGs: differentially expressed genes
KEGG: Kyoto Encyclopedia of Genes and Genomes
logFC: log2 fold change
DC: dendritic cells
GMP: granulocyte monocyte progenitor
BM: bone marrow
MAPK: mitogen-activated kinase-like protein
PI3K: phosphatidylinositol 3-kinase
JAK: Janus kinase
STAT: signal transducer and activator of transcription
IL-17: interleukin 17
TNF: tumor necrosis factor
JUNB: JunB proto-oncogene
FOSB: FosB proto-oncogene
FOSL1: FOS like 1
EGR1: early growth response 1.
